# Neurodegeneration in Patients with Type 2 Diabetes Mellitus without Diabetic Retinopathy

**DOI:** 10.1155/2019/1825819

**Published:** 2019-08-07

**Authors:** Elena Garcia-Martin, Marta Cipres, Isabel Melchor, Laura Gil-Arribas, Elisa Vilades, Vicente Polo, Maria Jesus Rodrigo, Maria Satue

**Affiliations:** ^1^Ophthalmology Department, Miguel Servet University Hospital, Zaragoza, Spain; ^2^Miguel Servet Ophthalmology Research Group (GIMSO), Aragon Health Research Institute (IIS Aragon), Zaragoza, Spain; ^3^Endocrinology Department, Miguel Servet University Hospital, Zaragoza, Spain

## Abstract

**Purpose:**

To evaluate neurodegeneration in patients with type 2 diabetes mellitus (DM2) without diabetic retinopathy and to assess the possible role of systemic vascular complications in retinal changes.

**Methods:**

Sixty eyes of 60 patients with DM2 and without any signs of diabetic retinopathy and 60 eyes of 60 healthy controls underwent retinal evaluation using Spectralis optical coherence tomography. Macular ganglion cell layer (GCL) and retinal nerve fiber layer (RNFL) were evaluated. Peripapillary RNFL thickness was assessed using Glaucoma and Axonal Analytics applications. Comparison between patients with the presence/absence of systemic vascular complications and different disease duration was made.

**Results:**

Macular GCL was reduced in patients compared to controls (*p* < 0.001). Differences in the macular RNFL thickness were only observed in the outer inferior sector (*p*=0.033). A reduction in the peripapillary RNFL (average, inferior, and inferotemporal thickness, *p* < 0.05 for all three) was observed in patients using both applications. Patients with chronic systemic vascular complications presented a reduction in the temporal RNFL (*p*=0.019) compared to patients without complications. The superotemporal RNFL thickness was thinner in patients with longer disease duration.

**Conclusions:**

Patients with type 2 DM without diabetic retinopathy and good metabolic control present neurodegeneration affecting neurons in the macular area and axons in different sectors of the optic disc. Systemic vascular complications contributed to further axonal damage in these patients, suggesting a possible role of subclinical ischaemia to retinal neurodegeneration in type 2 DM.

## 1. Introduction

Diabetic retinopathy in patients with diabetes mellitus (DM) was typically considered part of a vascular process. However, recent research suggested that retinal degeneration in DM might be caused not only by vasculopathy but also (and importantly) by neuropathy [1]. For some authors, neuropathy observed in the retina of DM patients might be just part of an underlying polyneuropathy [2, 3]; for others, however, neuropathic changes might precede microvascular alterations [4].

In the past decade, studies evaluating the neuroretina of DM patients using optical coherence tomography (OCT) technology tried to discern whether alterations affecting the retinal nerve fiber layer (RNFL) and other retinal layers preceded any vascular changes in these patients. Unfortunately, results were far from consistent.

Macular evaluation in DM patients might be biased by the presence of incipient edema, masking neuronal loss. The peripapillary area contains a higher density of axons (RNFL) compared to the macular area, and therefore, it should be considered an optimal location to evaluate axonal loss in these patients.

The purpose of the present study was to evaluate neurodegeneration in the macular (total thickness, ganglion cell layer thickness, and retinal nerve fiber layer thickness) and peripapillary (RNFL thickness) areas of patients with type 2 DM (DM2) without diabetic retinopathy (i.e., retinal vascular alterations or macular edema) and to assess the possible role of metabolic control, disease duration, and systemic vascular complications (ischaemia) on retinal changes in these patients.

## 2. Methods

We recruited sixty patients with type 2 DM and 60 age- and sex-matched healthy controls for the study. All procedures adhered to the tenets of the Declaration of Helsinki, and the local ethics committee (CEICA, ethics committee for scientific research in Aragon) approved the experimental protocol. All participants provided written informed consent to participate in the study.

The diagnosis of type 2 DM was established by an experienced endocrinologist based on the American Diabetes Association criteria [5] (Supplementary Table S1), and only patients without diabetic retinopathy (at least 12 months prior to recruitment) were eligible for the study. The International Clinical Disease Severity Scale for DR [6] was used for classification of any diabetic changes observed in our patients. This scale describes five stages that are recognized being the first of all “no apparent retinopathy.” As the name implies, there are no diabetic fundus changes. The second stage is “mild nonproliferative retinopathy” (NPDR), characterized by the presence of a few microaneurysms. There is a third stage, which is “moderate NPDR” and a fourth stage “severe NPDR.” The final stage is “proliferative diabetic retinopathy” (PDR). Only patients with no apparent retinopathy (based on methodical funduscopy and OCT scans) were included in our study.

The endocrine evaluation was performed by a trained specialist from the Endocrine and Nutrition Department of Miguel Servet University Hospital and provided demographic and disease-related parameters. Age at the time of diagnosis, disease duration, prescribed treatments (for DM2 and other comorbidities), presence of micro- and macrovascular complications (such as cardiovascular disease, cerebrovascular disease, diabetic neuropathy, or diabetic nephropathy), and presence of conditions related to chronic complications in DM (such as arterial hypertension, hypercholesterolemia, and obesity) were recorded. Endocrine variables such as glycosylated hemoglobin (HbA1C, most recent measured levels), microalbumin/creatinine ratio, and total, HDL (high-density lipoprotein), and LDL (low-density lipoprotein) cholesterol levels were obtained via blood analysis during routine checkups and recorded for the study. Arterial blood pressure and body mass index (BMI) were also checked. Subjects with arterial blood pressure levels of >140/90 mmHg or under current hypotensive treatment were classified as “hypertensive”; subjects with a calculated BMI of ≥25 were classified as “overweight/obese.” Smoking habit in patients and controls was also registered.

Inclusion criteria were confirmed type 2 DM diagnosis of at least 6 months, best-corrected visual acuity (BCVA) of 0.4 or higher (using a Snellen chart) in each eye to allow the performance of the protocol, and intraocular pressure less than 21 mmHg. Exclusion criteria were the presence or past history of diabetic retinopathy, confirmed by indirect funduscopy or retinography images, presence of significant refractive errors (≥5 diopters of spherical equivalent refraction or 3 diopters of astigmatism), intraocular pressure ≥21 mmHg, media opacifications, concomitant ocular diseases, including history of glaucoma or retinal pathology, and systemic conditions that could affect the visual system, including neurodegenerative disorders such as Parkinson's disease, multiple sclerosis, or dementia. Healthy controls had no record nor evidence of ocular or neurologic disease of any kind; their BCVA was >20/30 based on the Snellen scale. Only one eye per patient was selected at random and incorporated.

A complete ophthalmic evaluation that included pupillary, anterior segment, and funduscopic examination was performed in all subjects. We performed structural analysis of the retina using spectral domain (SD) optical coherence tomography (OCT) with the Spectralis OCT (Heidelberg, Germany), by three different applications: the Fast macular cube, for macular analysis, and the RNFL Glaucoma and RNFL-N Axonal Analytics, for peripapillary evaluation of the RNFL thickness. Part of this protocol was previously described by our team elsewhere [7].

A blue quality bar in the image indicates signal strength (range is 0–40, where 0 is categorized as poor quality and 40 as excellent). We included images with a score higher than 25 in analyses. Images were obtained using the image alignment eye-tracking software (TruTrack, Heidelberg Engineering) to acquire measurements of the nine ETDRS macular areas. Macular full retinal thickness, ganglion cell layer (GCL) thickness, and RNFL thickness were obtained using the Spectralis segmentation software. We also obtained measurements of the RNFL in the peripapillary area using the Glaucoma application (RNFL-G) and the Axonal Analytics application (RNFL-N). The RNFL thickness was measured around the disc with 16 average consecutive circular B-scans. The RNFL-N system places the temporal region of the scan in the center of the viewing window for better analysis of axonal loss in the papillomacular bundle (PMB), as seen in patients with neurologic diseases such as multiple sclerosis. The RNFL thickness graph for the RNFL-N scans reveals the scan results in the order of nasal-inferior-temporal-superior-nasal sectors. This protocol also provides 2 neuro-ophthalmologic parameters: the PMB thickness and the nasal/temporal (N/T) ratio. The additional PMB sector is displayed and classified in the pie chart of the thickness map. The parameter N/T ratio is defined as the mean RNFL thickness in the nasal quadrant divided by the mean RNFL thickness in the temporal quadrant. The parameters registered in the RNFL analysis were average thickness and thickness for six-disc sectors (superonasal, nasal, inferonasal, inferotemporal, temporal, and superotemporal).

### 2.1. Statistical Analysis

We performed all data analyses using SPSS software version 20.0 (SPSS Inc., Chicago, IL). All quantitative variables were expressed in mean and standard deviation (SD). All qualitative variables were expressed in number of cases and percentage. We used the Kolmogorov–Smirnov test to evaluate sample distribution. As a result of the normal distribution of the data, differences between assessments of DM2 patients and healthy subjects were compared using Student's *t*-test. The linear correlation between endocrine (age at the time of diagnosis, duration of the disease, HbA1C, total, HDL, and LDL cholesterol levels, systolic and diastolic arterial blood pressure, and BMI) and structural (full retinal thickness in the macular ETDRS areas and RNFL thickness in the peripapillary area) parameters was determined using Pearson's correlation coefficient.

Additionally, we performed a subclassification of subjects, dividing DM2 patients into 2 different groups depending on the presence/absence of some particular aspects: presence/absence of HbA1C levels >7 mmol/ml, disease duration ≥ or < 10 years, presence/absence of micromacrovascular complications, presence/absence of obesity, and presence/absence of hypertension. To avoid any confounding factor, the presence of smoking habit was analyzed and compared between controls and patients and also between subgroup of DM patients (smokers vs. nonsmokers). We compared results from the structural analysis of the retina between both groups using Student's *t*-test.

Values of *p* less than 0.05 were regarded as indicators of statistical significance. In order to avoid a high false-positive rate, the Bonferroni correction for multiple tests was calculated and the corrected *p* values were added to the previously calculated data (see tables).

## 3. Results

Sixty eyes of 60 patients with a mean age of 61.60 ± 9.40 years and 60 eyes of 60 healthy individuals with a mean age of 60.15 ± 7.05 were included in the study. There were 22 females (37%) and 38 (63%) males in the patient group and 36 females (60%) and 24 (40%) males in the control group. Patients and controls did not show significant differences in the four confounding factors considered: age (*p*=0.341), gender (*p*=0.220), intraocular pressure (*p*=0.132), and smoking habit (*p*=0.439). Disease duration in the group of patients was 12.62 years (SD = 7.29) (Supplementary Table S2). All demographic and endocrinology variables are displayed in Supplementary Table S1.

### 3.1. Structural Analysis

Structural measurements of the macular thickness provided by the Fast macular cube application showed significant retinal thinning in patients with DM2 compared with healthy subjects in all measured ETDRS areas (Table 1). Additionally, the ganglion cell layer was reduced in patients in all ETDRS sectors except in the center. The RNFL analysis revealed a significant reduction affecting the outer inferior sector (37.94 ± 6.08 *μ*m in patients vs. 40.29 ± 6.33 *μ*m in controls, *p*=0.033); however, no significant differences were observed in the other ETDRS areas.

The RNFL-G application demonstrated a significant RNFL thinning in DM patients in the average (95.93 ± 12.10 *μ*m in patients vs. 102.30 ± 9.22 *μ*m in controls, *p*=0.002), inferior (123.85 ± 18.91 *μ*m in patients vs. 135.50 ± 17.01 *μ*m in controls, *p*=0.001), temporal (68.42 ± 10.96 *μ*m vs. 74.17 ± 15.58 *μ*m, respectively, *p*=0.013), and inferotemporal (135.10 ± 20.44 *μ*m vs. 149.85 ± 21.17 *μ*m, *p* < 0.001) areas (Table 2). The same areas were significantly reduced when measured with the RNFL-N Axonal Analytics application (*p*=0.001). Additionally, a significant thinning in the average thickness (97.12 ± 12.29 *μ*m vs. 103.69 ± 9.55 *μ*m, *p*=0.001) of the RNFL was observed in patients with this application. The PMB was reduced in patients compared to controls (53.27 ± 8.31 *μ*m vs. 58.10 ± 10.50 *μ*m, *p*=0.006) (Table 2).

No significant correlations were observed between the endocrine variables and the retinal structural measurements obtained with Spectralis OCT (*p* > 0.05).

### 3.2. Subgroup Analysis

Diabetic patients were divided into 2 different groups depending on the presence or absence of a selected variable and then compared.

Patients with poor metabolic control (HbA1C > 7; *n* = 28) presented reduced retinal thickness in the nasal ETDRS macular sectors (inner nasal, *p*=0.022; outer nasal, *p*=0.013), in the superior RNFL sector (*p*=0.034 with Glaucoma application), and superonasal RNFL sector (*p*=0.003 with Glaucoma application and *p*=0.019 with Axonal Analytics application) (Table 3).

Patients with disease duration of at least 10 years (*n* = 38) presented a reduction in macular thickness in the outer superior (*p*=0.008) and outer temporal (*p*=0.004) areas of the ETDRS analysis compared to those with disease duration of less than 10 years (*n* = 22). The RNFL was thinner in the longer disease duration group in the superior (Axonal, *p*=0.019), inferior (Glaucoma, *p*=0.036), superotemporal (Glaucoma, *p*=0.029; Axonal, *p*=0.008), and inferotemporal (Glaucoma, *p*=0.012) sectors (Table 4).

Patients with the presence of chronic vascular complications of diabetes (*n* = 21) presented a reduction of the temporal sector of the RNFL thickness (Glaucoma, *p*=0.032; Axonal, *p*=0.019). The PMB was also thinner (*p*=0.019) in these patients compared to those without chronic vascular complications (*n* = 39) (Table 5).

Patients with obesity (*n* = 56) were compared with patients without obesity (*n* = 4). Macular external sectors (superior and temporal) were reduced in obese patients (308.25 ± 12.10 in normal weight vs. 290.73 ± 14.29 in obese patients, *p*=0.004; 301.50 ± 8.27 vs. 278.61 ± 13.11, *p* < 0.001, respectively). No significant reductions were observed in the peripapillary area between both groups.

The comparison between patients with/without hypertension, and smoker and nonsmoker patients did not show any significant differences in the structural variables.

## 4. Discussion

We evaluated retinal changes in 60 patients with type 2 DM without any sign of diabetic retinopathy using spectral domain OCT. Some authors suggested that a neuropathy (a totally different process from the typical vascular complications observed in diabetic retinopathy) might be causing ocular degeneration in patients with DM [1]. It was suggested that neuropathic changes might precede microvascular alterations in these patients [4]; however, the possible correlation between neuropathy and vasculopathy is still unknown. Funduscopic alterations such as microaneurysms and hemorrhages which are present at early stages in diabetic retinopathy might be preceded by other vascular alterations that are not observable in a routine exam. Thus, preclinical diabetic retinopathy might be caused by changes in the caliper of retinal vessels or in the regulation of retinal blood flow [8–10]. However, changes affecting retinal neurons have also been observed, such as increased apoptosis of the retinal ganglion cells and activation of the microglia [11–13] without signs of vascular changes.

OCT studies evaluating whether alterations affecting the RNFL and other retinal layers preceded any vascular changes in DM patients obtained opposite results [3,14–18], but a recent meta-analysis suggested this could be the case [19]. In our study, we observed an important reduction in macular thickness affecting all sectors of the ETDRS map. Early clinical studies using time domain OCT pointed to greater macular thickness in subjects with DM (with and without diabetic retinopathy) compared to controls [20]. More recent research suggested a similar thickness [21, 22] or significant reduction in the macular area in DM patients without diabetic retinopathy [23–25], being the latter supported by our own results. A possible explanation for the different obtained results concerning macular measurements is that macular thickness (affected by macular edema) in DM patients varies greatly with disease evolution and does not have a uniform behavior, and vascular alterations might mask other concomitant neurodegenerative processes affecting the same retinal area [26, 27]. Segmentation of the different macular layers in patients with DM presented different results among studies: recent studies [3, 17, 28, 29] demonstrated a reduction in the macular GCL in patients with DM (type 1 in Scarinci and Gundogan studies) without diabetic retinopathy, but some of them [28, 29] could not find any differences in the macular RNFL thickness between groups. Another recent study found a reduction in the macular RNFL and GCL in type 2 DM without diabetic retinopathy, but changes in the RNFL thickness were not significant after age correction for the diabetic group [30]. In our patients (type 2 DM without diabetic retinopathy), we observed a reduction in the GCL thickness affecting all sectors (except for the fovea), but barely no differences were observed in the macular RNFL thickness, supporting previously mentioned studies [28–30]. These results suggest that neurodegeneration (this is, degeneration of the ganglion cells) is present in early stages of DM, but axonal loss in the macular area might not be detectable simultaneously. Macular evaluation in DM patients without diabetic retinopathy, however, should be analyzed with caution. According to some authors, retinal damage caused by disease progression might trigger an immune-mediated response that leads to intra- and extracellular inflammation and macular edematous changes. Thus, macular thickness (or its individual layers) might not accurately reflect neurodegenerative changes in these patients.

The peripapillary area contains a higher density of axons (RNFL) compared to the macular area, and therefore, it should be considered an optimal location to evaluate axonal loss in DM patients. The peripapillary RNFL in our patients was assessed using 2 different OCT applications: the most frequently used Glaucoma application (RNFL-G) and the Axonal Analytics application (RNFL-N). The latter was specifically designed for the evaluation of neurodegenerative changes in the peripapillary area [31, 32]. In our patients, the RNFL was found to be reduced compared to controls, not only in average but especially in the inferotemporal sectors. The papillomacular bundle was also reduced in patients, suggesting a degeneration of the axonal path between the optic disc and the macular ganglion cells. These results also present anatomic correspondence with the distribution of the macular ganglion cells, whose axons correspond to the RNFL of the temporal and inferior sectors of the optic disc.

Our results support previous studies in which a reduction in the average RNFL thickness was observed in patients with DM2 without diabetic retinopathy [2, 3, 22, 33, 34]. Further RNFL damage was demonstrated by Carpineto et al. [35], who observed a reduction in the RNFL affecting all peripapillary quadrants. However, a reduction affecting the inferior quadrant was only demonstrated by Carpineto et al. [35] and Salvi et al. [3], which included the largest sample sizes. The affectation of the temporal sectors was barely reported in previous studies [35], and most important studies on this matter did not find differences affecting the temporal quadrant. Since the inferior sector is most frequently affected by hypoxia, it was reasonable to find a significant reduction in this quadrant. The reduction in the temporal areas might suggest the presence of neurodegeneration by different causes other than hypoxia, similarly to those observed in neurodegenerative diseases [36, 37].

One of the largest studies on OCT changes in DM patients concluded that retinal thinning in DM patients is caused by both vascular alterations and neurodegeneration, suggesting 2 different phenotypes for DM, one vascular (in which neurodegeneration is not present or has a minimum role) and the other (up to 60% of patients) more prone to neurodegeneration without vascular alterations [1]. Additionally, this study demonstrated that the association between OCT changes and alterations in the electroretinogram was stronger in cases where vasculopathy was present. However, it did not establish whether microvascular (subclinical) alterations played a significant role in the pathogenesis of the retinal changes in the neurodegenerative phenotype. In our study, patients without diabetic retinopathy but with chronic systemic vascular complications presented a further reduction of the temporal quadrant of the RNFL and the papillomacular bundle, suggesting that systemic ischaemia might indeed contribute to further damage in the neurodegenerative phenotype. Additionally, when metabolic control was assessed, patients with worse HbA1C levels presented a further reduction of the superior and nasal sectors; when disease duration was compared, a further reduction of the superior sectors was observed. These results might suggest that the presence of other different factors might also contribute to neuronal damage in these patients.

Our study presents different limitations: The sample selected for the present study includes subjects with different levels of disease duration and systemic vascular complications. However, we believe it is a fairly representative sample of the diabetic population in our society. Another limitation is the presence of different confounding factors associated with type 2 DM patients, such as hypertension and smoking habit. The presence of smoking habit was compared between patients and controls to avoid bias; additionally, subgroups of patients were compared to analyze the possible effect on structural alterations caused by these 2 variables. Since no differences were found, we assumed hypertension and smoking habit could be ruled out as confounding factors in our patients.

A secondary limitation to our study is the unavailability of new more precise devices such as OCT angiography. Additionally, a longitudinal follow-up of these patients would be needed to establish the influence of hypertension, obesity, systemic ischaemia, and so on in the development of DR.

Our results suggest that in patients with type 2 DM without diabetic retinopathy and good metabolic control, a neurodegeneration is present affecting especially neurons in the macular area and the RNFL in the temporal and inferior sectors of the optic disc. Additionally, the results obtained in our patients suggest that subclinical ischaemia, along with other metabolic factors, might be an important contributory factor to neurodegeneration in these patients. Further longitudinal studies are needed to establish the possible role of other systemic complications of DM (especially ischaemia) in the pathogenesis of retinal neurodegeneration.

## Figures and Tables

**Table 1:** Mean and standard deviation (in parentheses) of full retinal, ganglion cell layer, and retinal nerve fiber layer thickness (in microns) in the macular area, measured with Spectralis OCT in healthy controls and patients with DM2.

Macular parameters	Healthy	DM2	*p*
	Mean (±SD)	Mean (±SD)	
*Total thickness*			
Center	282.88 (±18.23)	275.62 (±19.18)	**0.035**
Inner superior	350.82 (±11.51)	335.38 (±14.77)	**<0.001** ^*∗*^
Inner nasal	355.23 (±11.94)	337.77 (±15.67)	**<0.001** ^*∗*^
Inner inferior	347.37 (±10.64)	331.67 (±14.79)	**<0.001** ^*∗*^
Inner temporal	338.13 (±9.66)	323.40 (±14.79)	**<0.001** ^*∗*^
Outer superior	302.20 (±14.76)	291.90 (±14.73)	**<0.001** ^*∗*^
Outer nasal	318.38 (±13.42)	307.33 (±14.29)	**<0.001** ^*∗*^
Outer inferior	289.48 (±13.05)	279.32 (±14.25)	**<0.001** ^*∗*^
Outer temporal	288.08 (±13.67)	280.13 (±14.03)	**0.002** ^*∗*^

*GCL thickness*			
Center	16.53 (±6.01)	16.63 (±4.18)	0.908
Inner superior	54.17 (±4.23)	49.59 (±5.99)	**<0.001** ^*∗*^
Inner nasal	53.90 (±4.78)	48.00 (±6.48)	**<0.001** ^*∗*^
Inner inferior	54.29 (±3.98)	48.96 (±5.98)	**<0.001** ^*∗*^
Inner temporal	49.44 (±4.18)	43.63 (±6.49)	**<0.001** ^*∗*^
Outer superior	35.97 (±3.22)	33.56 (±3.22)	**<0.001** ^*∗*^
Outer nasal	39.16 (±3.28)	36.50 (±3.76)	**<0.001** ^*∗*^
Outer inferior	33.38 (±3.24)	31.85 (±3.71)	**0.014**
Outer temporal	36.38 (±3.76)	33.91 (±4.68)	**0.001** ^*∗*^
Total volume	1.11 (±0.10)	1.03 (±0.75)	**<0.001** ^*∗*^

*RNFL thickness*			
Center	13.12 (±1.67)	13.02 (±1.66)	0.752
Inner superior	23.73 (±2.89)	23.89 (±2.89)	0.782
Inner nasal	21.25 (±2.35)	20.95 (±2.68)	0.502
Inner inferior	25.70 (±3.10)	24.67 (±3.09)	0.065
Inner temporal	17.55 (±1.61)	17.64 (±1.56)	0.742
Outer superior	37.09 (±4.99)	36.69 (±6.76)	0.700
Outer nasal	49.34 (±7.45)	47.45 (±7.10)	0.139
Outer inferior	40.29 (±6.33)	37.94 (±6.08)	**0.033**
Outer temporal	19.31 (±1.64)	19.39 (±1.63)	0.781
Total volume	0.92 (±0.10)	0.89 (±0.10)	0.137

Bold letters indicate statistical significance (*p* < 0.05). Asterisks mark statistical significance using Bonferroni correction (*p* < 0.005). DM, diabetes mellitus; SD, standard deviation; GCL, ganglion cell layer; RNFL, retinal nerve fiber layer.

**Table 2:** Structural measurements obtained with Spectralis OCT in healthy controls and patients with type 2 diabetes mellitus (DM2) without diabetic retinopathy.

Peripapillary measurements	Healthy	DM2	*p*
	Mean (±SD)	Mean (±SD)	
*RNFL Glaucoma*			
Average	102.30 (±9.22)	95.93 (±12.10)	**0.002** ^*∗*^
Superior	123.42 (±16.58)	118.98 (±19.37)	0.180
Nasal	75.92 (±15.29)	72.48 (±14.84)	0.214
Inferior	135.50 (±17.01)	123.85 (±18.91)	**0.001** ^*∗*^
Temporal	74.17 (±15.58)	68.22 (±9.73)	**0.013**
Superotemporal	137.27 (±16.77)	132.10 (±21.76)	0.148
Inferotemporal	149.85 (±21.17)	135.10 (±20.44)	**<0.001** ^*∗*^
Superonasal	109.57 (±22.76)	105.85 (±22.89)	0.374
Inferonasal	121.15 (±24.61)	112.60 (±23.81)	0.055

*RNFL Axonal Analytics*			
Average	103.69 (±9.55)	97.12 (±12.29)	**0.001** ^*∗*^
Superior	125.63 (±17.02)	120.18 (±18.95)	0.102
Nasal	77.98 (±14.18)	74.27 (±15.38)	0.173
Inferior	136.97 (±17.41)	125.50 (±20.14)	**0.001** ^*∗*^
Temporal	73.98 (±13.33)	68.42 (±10.96)	**0.014**
Superonasal	112.59 (±22.79)	107.53 (±21.35)	0.214
Inferonasal	123.68 (±23.26)	114.65 (±24.80)	**0.043**
Superotemporal	138.66 (±18.20)	132.82 (±22.22)	0.120
Inferotemporal	150.27 (±22.38)	136.35 (±21.08)	**0.001** ^*∗*^
Papillomacular bundle	58.10 (±10.50)	53.27 (±8.31)	**0.006**
Nasal/temporal index	1.10 (±0.32)	1.11 (±0.29)	0.793

Bold letters indicate *p* < 0.05. Asterisks mark significant values according to Bonferroni corrections for multiple comparisons. DM2, type 2 diabetes mellitus; RNFL: retinal nerve fiber layer; SD, standard deviation.

**Table 3:** Structural measurements obtained with Spectralis OCT in patients with type 2 diabetes mellitus (DM2) with glycated hemoglobin (HbA1C) ≤7% and >7%, respectively.

	HbA1C ≤ 7		HbA1C > 7		*p*
	Mean	SD	Mean	SD	
*Macular thickness*					
Central	279.03	14.53	271.71	23.06	0.142
Inner superior	338.66	14.24	331.64	14.73	0.066
Inner nasal	342.06	13.73	332.86	16.52	**0.022**
Inner inferior	334.03	13.83	328.96	15.63	0.188
Inner temporal	325.78	13.39	320.68	16.06	0.185
Outer superior	294.63	13.73	288.79	15.45	0.127
Outer nasal	311.56	12.84	302.50	14.55	**0.013**
Outer inferior	281.31	12.96	277.04	15.51	0.249
Outer temporal	282.81	13.66	277.07	14.05	0.115

*pRNFL Glaucoma*					
Average	98.19	9.15	93.36	14.53	0.124
Superior	123.91	16.59	113.34	21.03	**0.034**
Nasal	75.44	11.89	69.11	17.22	0.100
Inferior	125.58	14.87	121.88	22.80	0.454
Temporal	67.44	10.52	69.11	8.85	0.512
Superotemporal	134.00	17.98	129.93	25.58	0.474
Inferotemporal	136.84	15.54	133.11	25.05	0.485
Superonasal	113.81	22.11	96.75	20.57	**0.003** ^*∗*^
Inferonasal	114.31	22.63	110.64	25.36	0.556

*pRNFL Axonal Analytics*					
Average	99.69	9.23	94.18	14.67	0.083
Superior	124.39	15.78	115.36	21.29	0.065
Nasal	77.19	12.85	70.93	17.48	0.117
Inferior	128.47	16.06	122.11	23.83	0.225
Temporal	68.44	11.41	68.39	10.62	0.988
Superonasal	113.50	20.12	100.71	20.99	**0.019**
Inferonasal	117.38	24.35	111.54	25.38	0.367
Superotemporal	135.28	19.50	130.00	25.05	0.363
Inferotemporal	139.56	15.96	132.68	25.54	0.210
Papillomacular bundle	52.25	8.48	54.43	8.11	0.315
Nasal/temporal index	1.16	0.28	1.05	0.30	0.147

Bold letters indicate *p* < 0.05. Asterisks mark significant values according to Bonferroni corrections for multiple comparisons. DM2, type 2 diabetes mellitus; pRNFL: peripapillary retinal nerve fiber layer; SD, standard deviation.

**Table 4:** Structural measurements obtained with Spectralis OCT in patients with type 2 diabetes mellitus with disease duration of <10 and ≥10 years, respectively.

	Disease duration <10 years		Disease duration ≥10 years		*p*
	Mean	SD	Mean	SD	
*Macular thickness*					
Central	274.77	16.12	276.11	20.94	0.798
Inner superior	338.91	13.57	333.34	15.22	0.161
Inner nasal	339.64	15.13	336.68	16.07	0.487
Inner inferior	334.27	13.22	330.16	15.60	0.303
Inner temporal	327.09	10.51	321.26	16.54	0.143
Outer superior	298.45	12.53	288.11	14.71	**0.008**
Outer nasal	309.45	13.23	306.11	14.91	0.386
Outer inferior	282.95	12.05	277.21	15.12	0.133
Outer temporal	286.82	11.24	276.26	14.15	**0.004** ^*∗*^

*pRNFL Glaucoma*					
Average	98.91	9.77	94.21	13.08	0.149
Superior	123.36	15.85	116.43	20.92	0.184
Nasal	73.36	12.53	71.97	16.16	0.730
Inferior	130.52	16.29	119.99	19.43	**0.036**
Temporal	68.18	11.18	68.24	8.95	0.983
Superotemporal	140.09	19.21	127.47	22.03	**0.029**
Inferotemporal	143.73	15.05	130.11	21.63	**0.012**
Superonasal	106.64	20.03	105.39	24.65	0.842
Inferonasal	117.32	27.17	109.87	21.54	0.246

*pRNFL Axonal Analytics*					
Average	100.23	10.07	95.32	13.20	0.137
Superior	127.66	17.14	115.84	18.79	**0.019**
Nasal	76.27	13.61	73.11	16.38	0.447
Inferior	130.16	16.33	122.80	21.80	0.175
Temporal	66.95	10.62	69.26	11.20	0.436
Superonasal	112.68	21.97	104.55	20.68	0.157
Inferonasal	118.82	27.70	112.24	23.00	0.326
Superotemporal	142.64	21.07	127.13	21.10	**0.008**
Inferotemporal	141.50	14.36	133.37	23.80	0.151
Papillomacular bundle	51.05	8.39	54.55	8.10	0.116
Nasal/temporal index	1.16	0.26	1.08	0.31	0.301

Bold letters indicate *p* < 0.05. Asterisks mark significant values according to Bonferroni corrections for multiple comparisons. DM2, type 2 diabetes mellitus; pRNFL: peripapillary Rretinal nerve fiber layer; SD, standard deviation.

**Table 5:** Structural measurements obtained with Spectralis optical coherence tomography in patients with type 2 diabetes mellitus, with and without vascular complications.

	Without complications		With complications		*p*
	Mean	SD	Mean	SD	
*Macular thickness*					
Central	278.23	15.19	270.76	24.68	0.152
Inner superior	336.41	13.48	333.48	17.11	0.468
Inner nasal	338.87	15.04	335.71	16.95	0.461
Inner inferior	332.49	15.11	330.14	14.41	0.563
Inner temporal	323.90	14.89	322.48	14.93	0.726
Outer superior	291.49	14.86	292.67	14.82	0.770
Outer nasal	306.74	14.65	308.43	13.90	0.667
Outer inferior	280.00	15.68	278.05	11.36	0.617
Outer temporal	278.85	14.11	282.52	13.89	0.337

*pRNFL Glaucoma*					
Average	97.36	14.27	93.29	5.80	0.217
Superior	121.78	22.13	113.76	11.53	0.127
Nasal	72.79	16.01	71.90	12.71	0.827
Inferior	124.49	22.29	122.67	10.40	0.725
Temporal	70.18	9.95	64.57	8.36	**0.032**
Superotemporal	135.15	24.58	126.43	14.02	0.140
Inferotemporal	135.54	23.83	134.29	12.34	0.823
Superonasal	108.41	24.43	101.10	19.39	0.241
Inferonasal	113.44	26.71	111.05	17.68	0.714

*pRNFL Axonal Analytics*					
Average	98.59	14.51	94.38	5.78	0.208
Superior	122.86	21.36	115.19	12.35	0.136
Nasal	74.54	16.64	73.76	13.08	0.854
Inferior	125.86	23.62	124.83	11.65	0.853
Temporal	70.82	11.14	63.95	9.28	**0.019**
Superonasal	109.85	23.38	103.24	16.60	0.256
Inferonasal	114.85	27.77	114.29	18.68	0.934
Superotemporal	135.87	24.28	127.14	16.87	0.148
Inferotemporal	136.87	24.91	135.38	11.41	0.796
Papillomacular bundle	55.23	8.19	49.62	7.40	**0.011**
Nasal/temporal index	1.08	0.32	1.17	0.23	0.236

Bold letters indicate *p* < 0.05. Asterisks mark significant values according to Bonferroni corrections for multiple comparisons. DM2, type 2 diabetes mellitus; pRNFL: peripapillary retinal nerve fiber layer; SD, standard deviation.

## Data Availability

The data used to support the findings of this study are available from the corresponding author upon request.

## Supplementary Materials

Supplementary Materials Supplementary Table 1: diagnostic criteria for diabetes mellitus according to the American Diabetes Association. Supplementary Table 2: demographic and epidemiologic data of patients with type 2 diabetes mellitus included in the study.
