# Supplementary material for: Neurodegeneration in Patients with Type 2 Diabetes Mellitus without Diabetic Retinopathy
Source: J Ophthalmol. 2019 Aug 7;2019:1825819. doi: 10.1155/2019/1825819 (PMC6702840; doi:10.1155/2019/1825819)
Supplement: Supplementary Materials — Supplementary Table 1: diagnostic criteria for diabetes mellitus according to the American Diabetes Association. Supplementary Table 2: demographic and epidemiologic data of patients with type 2 diabetes mellitus included in the study. [file 1825819.f1.zip › 1825819.f1/Suppl table 1.docx]

| Glycosylated hemoglobin (HbA1C) ≥ 6.5%. (The HbA1C must have been determined with a method certified by the NGSP (National Glycohemoglobin Standardization Program), and with traceability to the reference method of the DCCT (Diabetes Control and Complications Trial) *. |
| --- |
| Fasting plasma glucose ≥ 126 mg / dl. Defining fasting as the absence of caloric intake for at least 8 hours *. |
| Glycemia ≥ 200 mg / dl 2 hours after the Oral Glucose Tolerance Test (OGTT) (performed with a solution containing 75 g of glucose) *. |
| Random blood glucose ≥ 200 mg / dl together with the presence of classic symptoms of diabetes (polydipsia, polyuria, weight loss ...) |

**Supplementary table 1:** Diagnostic criteria for Diabetes Mellitus according to the American Diabetes Association.
