# Supplementary material for: Neurodegeneration in Patients with Type 2 Diabetes Mellitus without Diabetic Retinopathy
Source: J Ophthalmol. 2019 Aug 7;2019:1825819. doi: 10.1155/2019/1825819 (PMC6702840; doi:10.1155/2019/1825819)
Supplement: Supplementary Materials — Supplementary Table 1: diagnostic criteria for diabetes mellitus according to the American Diabetes Association. Supplementary Table 2: demographic and epidemiologic data of patients with type 2 diabetes mellitus included in the study. [file 1825819.f1.zip › 1825819.f1/Suppl table 2.docx]

|  | **Mean±SD** |
| --- | --- |
| Disease duration (years) | 12.62±7.29 |
| Age at diagnosis (years) | 50,50±10.57 |
| Glycated hemoglobin (%) | 7.33±0.89 |
| mycroalbuminuria/creatinin index | 18.23±21.20 |
|  | **Number of patients (%)** |
| Presence of hypertension | 49 (81.87%) |
| Presence of dyslipemia | 57 (95%) |
| Presence of vascular complications |  |
| Ischaemic cardiopathy | 8 (13.30%) |
| Cerebrovascular disease | 2 (3.30%) |
| Peripheric vasculopathy | 0 (0%) |
| Diabetic neuropathy | 5 (8.33%) |
| Diabetic nephropathy | 8 (13.30%) |
| Treatment |  |
| No insuline (only oral antidiabetic agents) | 43 (71.60%) |
| Insuline | 17 (28.40%) |

**Supplementary table 2:** Demographic and epidemiologic data of patients with type 2 diabetes mellitus included in the study.

Abbreviations: SD, standard deviation
